# Actin-binding protein filamin B regulates the cell-surface retention of endothelial sphingosine 1-phosphate receptor 1

**DOI:** 10.1016/j.jbc.2023.104851

**Published:** 2023-05-21

**Authors:** Xian Zhao, Keisuke Kiyozuka, Akimitsu Konishi, Reika Kawabata-Iwakawa, Yoji Andrew Minamishima, Hideru Obinata

**Affiliations:** 1 Department of Biochemistry, Gunma University Graduate School of Medicine, Maebashi, Gunma, Japan; 2 Division of Integrated Oncology Research, Gunma University Initiative for Advanced Research, Gunma University, Maebashi, Gunma, Japan; 3 Education and Research Support Center, Gunma University Graduate School of Medicine, Maebashi, Gunma, Japan

**Keywords:** sphingosine 1-phosphate (S1P), sphingolipid, filamin, G protein–coupled receptor (GPCR), receptor internalization, cell migration, endothelial cell, proximity labeling proteomics

## Abstract

Sphingosine 1-phosphate receptor 1 (S1PR1) is a G protein–coupled receptor essential for vascular development and postnatal vascular homeostasis. When exposed to sphingosine 1-phosphate (S1P) in the blood of ∼1 μM, S1PR1 in endothelial cells retains cell-surface localization, while lymphocyte S1PR1 shows almost complete internalization, suggesting the cell-surface retention of S1PR1 is endothelial cell specific. To identify regulating factors that function to retain S1PR1 on the endothelial cell surface, here we utilized an enzyme-catalyzed proximity labeling technique followed by proteomic analyses. We identified Filamin B (FLNB), an actin-binding protein involved in F-actin cross-linking, as a candidate regulating protein. We show FLNB knockdown by RNA interference induced massive internalization of S1PR1 into early endosomes, which was partially ligand dependent and required receptor phosphorylation. Further investigation showed FLNB was also important for the recycling of internalized S1PR1 back to the cell surface. FLNB knockdown did not affect the localization of S1PR3, another S1P receptor subtype expressed in endothelial cells, nor did it affect localization of ectopically expressed β2-adrenergic receptor. Functionally, we show FLNB knockdown in endothelial cells impaired S1P-induced intracellular phosphorylation events and directed cell migration and enhancement of the vascular barrier. Taken together, our results demonstrate that FLNB is a novel regulator critical for S1PR1 cell-surface localization and thereby proper endothelial cell function.

Cardiovascular diseases remain one of the most common causes of death worldwide (1), and vascular injury and inflammation account for the major risks (2). Vascular homeostasis is regulated by many factors, among which sphingosine 1-phosphate (S1P) signaling has been acknowledged to be essential for vascular development and postnatal vascular homeostasis (3, 4). S1P is a bioactive lipid mediator participating in various cellular functions such as proliferation, migration, adhesion, and inflammatory responses in many types of cells, especially in the immune and vascular systems (4). Genetic loss-of-function studies in mice have identified critical roles of S1P in embryonic development and physiological processes of multiple organ systems. For example, mice that lack sphingosine kinases, key enzymes for S1P production, are embryonic lethal due to disturbed neurogenesis and angiogenesis at early stages of development (5). Postnatally, S1P signaling maintains vascular homeostasis by enhancing endothelial cell barrier function and regulating vascular tone, which is fundamental for maintaining blood flow and systemic blood pressure (6, 7).

S1P exerts its bioactive functions by acting on high-affinity G protein–coupled receptors (S1PR1-5). S1PR1-3 are widely distributed with high expression levels in the cardiovascular and immune systems. S1PR4 and S1PR5 show limited expression in the lymphatic and nervous systems, respectively (8). S1PR1 is the predominant receptor in the endothelium, through which S1P regulates vascular homeostasis, and S1PR3 is expressed both in the endothelial and smooth muscle layers of arteries. Global deletion or endothelial-specific deletion of S1PR1 in mouse embryos results in lethality between E12.5 and E14.5 due to hemorrhagic vascular leak (3, 9). However, single deletion of either S1PR2 or S1PR3 does not result in embryonic lethality, which indicates the critical role of S1PR1 in vascular maturation (10).

In physiological conditions, activation of endothelial S1PR1 signaling inhibits sprouting angiogenesis, strengthens the adherens junctions between endothelial cells, and maintains vascular homeostasis (11). Loss of endothelial S1PR1 in mice causes hypersprouting phenotypes of endothelial cells located in the leading front of the neovascularization, with destabilized adherens junctions, enhanced vascular leak, and disturbed blood flow (12). Upon S1P binding, S1PR1 enhances the endothelial barrier *via* activating Gαi, which in turn induces intracellular Ca^2+^ mobilization (13) as well as activation of small GTPases Rac1 and Cdc42 (14), thereby stabilizing vascular endothelial-cadherin (VE-cadherin) junctions. Cell migration and angiogenesis induced by S1P-S1PR1 signaling have been ascribed to the activation of PI3K/Akt/endothelial NO synthase pathway (15), while prosurvival/antiapoptotic signaling from S1PR1 is closely correlated with increased activation of ERK1/2 (16). The functional importance of each pathway is dependent on the specific cellular contexts. However, the S1P signaling axis is multifaceted, depending on the carrier proteins, receptor subtypes, downstream effectors, and other factors (17, 18, 19).

Vascular inflammation is an integrated and complex response and involves many cell types and numerous mediators (20). Although the molecular mechanisms have not been completely clarified, S1PR1 signaling regulates the inflammatory status of vascular endothelial cells. Specific deletion of S1PR1 in endothelial cells resulted in increased expressions of proinflammatory factors such as vascular cell adhesion molecule 1 and intercellular adhesion molecule-1, and endothelial S1PR1-deleted mice with a high-fat diet showed more severe atherosclerotic lesions in the descending aorta (21). In line with this, endothelial S1PR1 showed intracellular localization in the inflammation-prone areas, in contrast to cell-surface accumulation under the laminar flow (12), which further confirms the importance of S1PR1 signaling in regulating vascular inflammation. These observations indicate that proper S1PR1 localization and signaling are required to maintain vascular homeostasis and that disturbed S1PR1 signaling due to the receptor internalization predisposes endothelial cells to an inflammatory state.

S1P–S1PR1 signaling also plays a critical role in the immune system. The S1P gradient (low in lymphoid tissues while high in lymph and blood) regulates lymphocyte trafficking. Dysregulation of this gradient results in a substantial decrease in circulating lymphocytes due to defects in lymphocyte egress from lymphoid tissues to lymph (22). Fingolimod, a synthetic S1P analogue, was approved by the US Food and Drug Administration in 2010 as the first oral medicine for the treatment of relapsing-remitting multiple sclerosis (23, 24). Fingolimod induces sustained internalization and degradation of S1PR1 both in lymphocytes and endothelial cells, leading to decreased expression of S1PR1 (25). One of the most severe adverse effects of fingolimod is macular edema due to impaired vascular barrier function likely caused by the downregulation of endothelial S1PR1 (26). S1PR1 shows cell-surface residency in endothelial cells while it is almost completely internalized in lymphocytes when exposed to the same concentration of S1P in blood (27, 28). In lymphocytes, CD69 induces S1PR1 conformational change and subsequent endocytosis and degradation (29). However, those factors involved in sustaining the cell-surface retention of endothelial S1PR1 remain unknown.

In order to discover factors that could potentially regulate S1PR1 cell-surface retention in an endothelium-specific manner, we took advantage of a TurboID system (30) to label proteins near S1PR1 with biotin, followed by purification of the labeled proteins and a mass spectrometry–based protein identification, targeting proteins interacting with cell surface but not with intracellular S1PR1. As a result, we found that filamin B (FLNB) not only maintains cell-surface retention of endothelial S1PR1 but also facilitates the recycling back of endocytosed S1PR1 to the cell surface, thereby sustaining proper endothelial functions through the S1P–S1PR1 signaling system.

## Results

### S1PR1 mutant S1PR1-TM4 shows intracellular localization in endothelial cells

**Figure 1:**
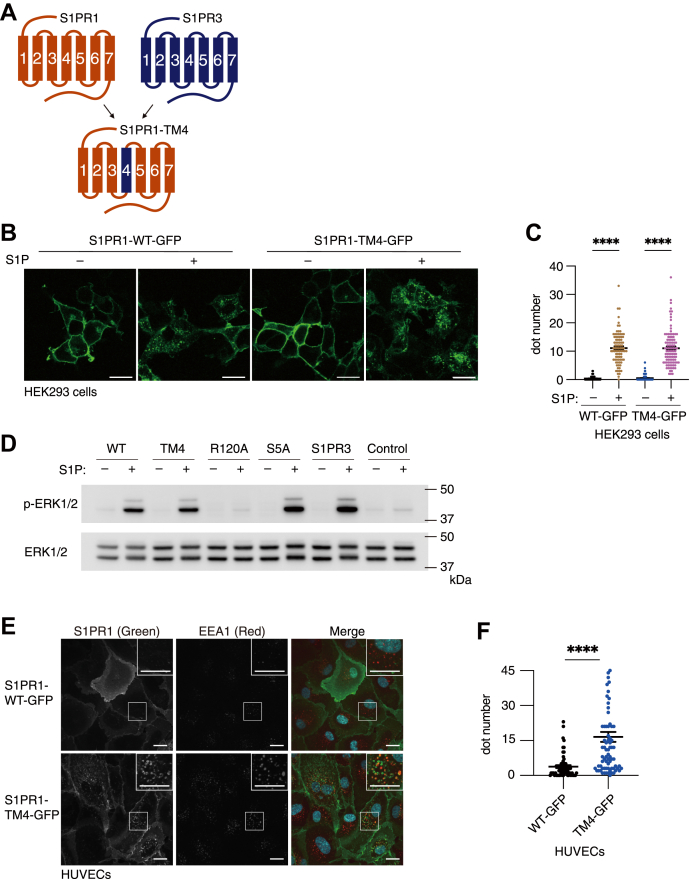
**S1PR1 mutant S1PR1-TM4 shows intracellular localization in endothelial cells.***A*, cartoon illustrating the wildtype S1PR1, S1PR3, and S1PR1-TM4 chimeric protein. *B*, representative images of GFP-tagged S1PR1-WT or -TM4 expressed in HEK293 cells with or without 200 nM S1P stimulation. The scale bar represents 20 μm. *C*, quantification of the fluorescent dot numbers of internalized GFP-tagged S1PR1 in (*B*). Data represent mean ± SEM. ∗∗∗∗*p* < 0.001 in Student’s *t* test. *D*, detection of ERK1/2 phosphorylation mediated by various mutants of S1PR1 and wildtype S1PR3 (all GFP-tagged) expressed in CHO cells after 100 nM S1P stimulation for 5 min. WT, wildtype S1PR1; TM4, S1PR1-TM4; R120A, S1P binding-deficient mutant used in Figure 5; S5A, internalization-deficient mutant used in Figure 5; S1PR3, wildtype S1PR3; Control, vector-infected control. *E*, representative images of human umbilical vein endothelial cells (HUVECs) showing the localization of GFP-tagged S1PR1-WT or -TM4. The cells were fixed and stained with anti-EEA1 antibodies and Alexa Fluor 568–conjugated secondary antibody. The images in the white rectangles are enlarged in the insets. Yellow puncta in the merge image indicate colocalization. The scale bar represents 20 μm. *F*, quantification of the fluorescent dot numbers of internalized GFP-tagged S1PR1 in (*E*). Data represent mean ± SEM. ∗∗∗∗*p* < 0.0001 in Student’s *t* test. Data are representatives from at least two independent experiments. Dot numbers were counted in more than 50 cells in each condition from the three sets of independent experiments in (*C*) and (*F*).

A previous study has shown that CD69 binds to the transmembrane helix 4 (TM4) of S1PR1 in lymphocytes to induce S1PR1 endocytosis and degradation (29), which inhibits S1PR1 signaling and lymphocyte egress from lymphoid organs (31). However, CD69 is not expressed in endothelial cells, and we assumed that other factors might regulate S1PR1 endocytosis through TM4 in an endothelium-specific manner. To find out such factors, we constructed the S1PR1 mutant in which TM4 is replaced by that of S1PR3 (abbreviated as S1PR1-TM4 hereafter, Figure 1*A*) (29). When expressed in HEK293 cells, both S1PR1-WT and -TM4 (GFP-tagged) showed cell-surface localization and became internalized after S1P stimulation (Fig. 1, *B* and *C*). Also, S1PR1-TM4 induced ERK1/2 phosphorylation after S1P stimulation to the same extent as WT in CHO cells (Fig. 1*D*). These data demonstrate that the S1PR1-TM4 mutant maintains normal S1PR1-dependent Gαi activation in HEK293 and CHO cells. However, when expressed in human umbilical vein endothelial cells (HUVECs), S1PR1-TM4 showed markedly increased intracellular localization in vesicle-like structures even without S1P stimulation (Fig. 1, *E* and *F*). Immunostaining of EEA1 (an early endosome marker) revealed that most of the internalized S1PR1-TM4 was found in early endosomes (Fig. 1*E*), indicating that the increased intracellular localization of S1PR1-TM4 was not due to the accumulation of misfolded protein but due to enhanced endocytosis. S1PR1-TM4 showed increased intracellular localization also in other endothelial cells such as human dermal microvascular endothelial cells (Fig. S1*A*) and murine embryonic endothelial cells (Fig. S1*B*). These data suggest that some proteins interact with the TM4 domain of S1PR1 and enable cell-surface localization of S1PR1 in an endothelial cell–specific manner.

**Figure 2:**
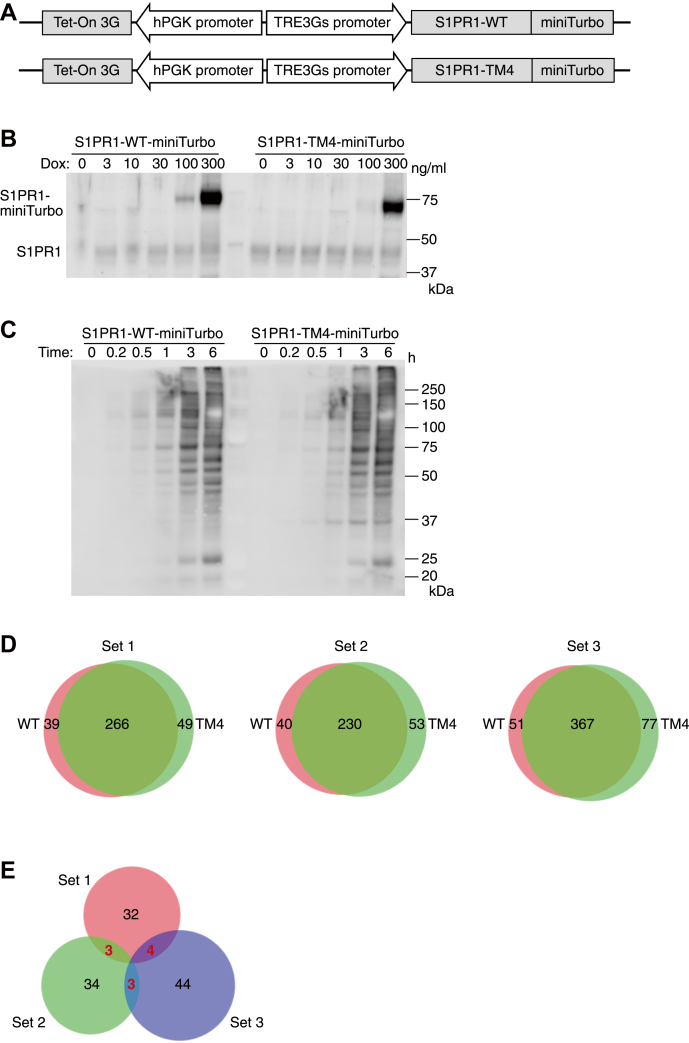
**Identification of proximal and interacting proteins of S1PR1 in human umbilical vein endothelial cells (HUVECs).***A*, diagrams of the expression cassettes used for the expression of a biotin ligase, miniTurbo. miniTurbo was fused to the C terminus of S1PR1-WT/-TM4. Expression was under the control of a TRE3Gs promoter and doxycycline-dependent transactivator protein Tet-On 3G. *B*, Western blot of S1PR1-WT/-TM4-miniTurbo expressions in HUVECs induced by different concentrations of doxycycline (Dox) for 24 h. The cells were lysed, and the ligase-tagged S1PR1 were identified by anti-S1PR1 antibody. *C*, Western blot of the biotinylated proteins by S1PR1-WT/-TM4- miniTurbo expression in HUVECs. The cells were treated with 100 ng/ml doxycycline for 24 h, then treated with 500 μM biotin for indicated times. The cells were lysed, and the biotinylated proteins were visualized by streptavidin-HRP. *D*, Venn diagrams depicting the protein numbers that were identified in the shot-gun proteomic analyses of the biotinylated proteins in three sets of independent experiments. WT, S1PR1-WT-miniTurbo (*left*); TM4, S1PR1-TM4-miniTurbo (*right*). *E*, Venn diagram depicting the protein numbers that were detected in WT but not in TM4 in the three sets of independent experiments. The protein numbers overlapping in the two sets are shown in *red*.

To find out factors that are important for the cell-surface localization of S1PR1 in endothelial cells, we compared the proximal S1PR1-WT interactome with that of S1PR1-TM4 using an enzyme-catalyzed proximity labeling system, TurboID (30), that was fused to the C terminus of S1PR1. TurboID or miniTurbo (mutated TurboID that shows slower kinetics but higher specificity) was fused to the C terminus of S1PR1-WT/-TM4 (Fig. 2*A* for miniTurbo and S2A for TurboID) and expressed in HUVECs. To minimize nonspecific biotinylation, the expression of the enzyme-tagged S1PR1-WT/-TM4 was controlled to the same extent as endogenous S1PR1 by a tetracycline-inducible system. Doxycycline titration analysis was performed, and 100 ng/ml was selected for the downstream analysis (Figs. 2*B* and S2*B*). After incubation with biotin (substrate for TurboID and miniTurbo) for indicated times, the biotinylated proteins were visualized by streptavidin-HRP antibody (Figs. 2*C* and S2*C*). Although TurboID catalyzed earlier and higher protein biotinylation than miniTurbo, it also gave rise to more background (30). Therefore, we prioritized the labeling specificity and chose the miniTurbo samples incubated with biotin for 3 h for further analysis.

After the purification of biotin-labeled proteins by using Tamavidin 2-REV magnetic beads, the proteins were digested on beads by trypsin and LysC and applied to a shot-gun proteomic analysis. We performed triplicate measurements for three sets, and around 300 proteins were commonly identified both in WT and TM4 in the triplicate samples in each set (Fig. 2*D*). To narrow down candidate proteins associated with cell-surface S1PR1-WT but not with internalized S1PR1-TM4, we selected the proteins identified only in the WT samples, which gave 39, 40, and 51 proteins in set 1 to 3, respectively. Then, we further selected the proteins commonly found at least in the two sets of the experiments (Fig. 2*E*), and 10 proteins matched these criteria (Table 1). Judging from annotated functions and localization of these proteins, we selected six candidate proteins for further analysis, which were Perlecan (HSPG2), integrin alpha-2 (ITGA2), filamin B (FLNB), RhoGEF and PH domain-containing protein 5 (FGD5), Elongation factor 1-gamma (EF1G), and Ras GTPase-activating protein-binding protein 2 (G3BP2).

**Table 1:** Ten proteins identified primarily in S1PR1-WT not in -TM4

No	Gene ID	Full name
1	HSPG2	Perlecan
2	ITGA2	Integrin alpha-2
3	FLNB	Filamin B
4	FGD5	RhoGEF and PH domain–containing protein 5
5	EF1G	Elongation factor 1-gamma
6	G3BP2	Ras GTPase-activating protein-binding protein 2
7	PRAF2	PRA1 family protein 2
8	PDIA3	Protein disulfide-isomerase A3
9	SEPT2	Septin-2
10	VPS13C	Vacuolar protein sorting-associated protein 13C

### FLNB knockdown induces S1PR1 internalization in HUVECs

**Figure 3:**
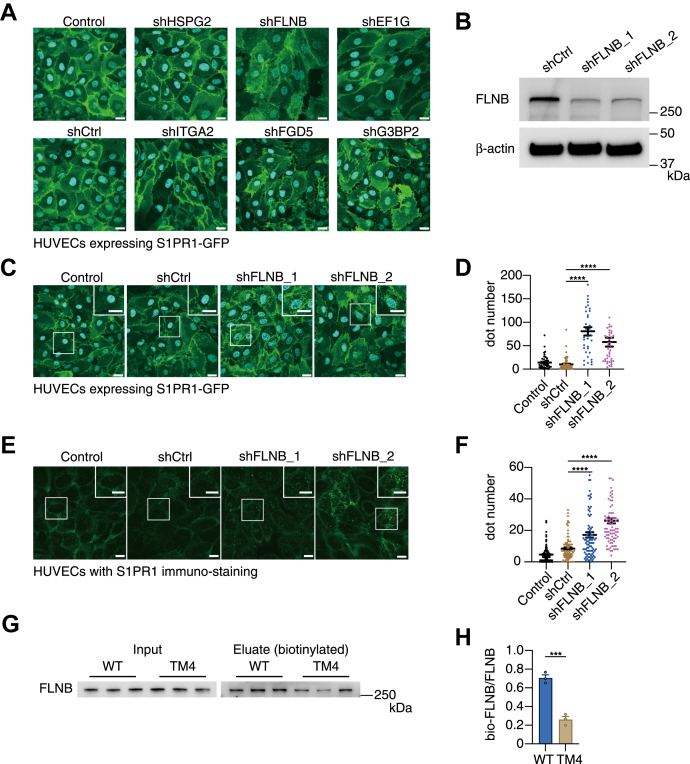
**FLNB knockdown induces S1PR1 internalization in human umbilical vein endothelial cells (HUVECs).***A*, representative images of HUVECs showing the localization of GFP-tagged S1PR1 in HUVECs with knockdown of the candidate proteins by shRNA. HUVECs expressing S1PR1-GFP was infected with lentivirus to express shRNA for the candidate proteins or nontarget control (shCtrl) for 48 h, fixed with 4% paraformaldehyde, stained with DAPI for nuclei, then imaged under a confocal microscope. The scale bar represents 20 μm. *B*, expression levels of FLNB protein after knockdown by two different shRNA in HUVECs. *C*–*F*, representative images of HUVECs showing the localization of GFP-tagged S1PR1 (*C*) or endogenous S1PR1 (*E*) in HUVECs with or without FLNB knockdown. HUVECs with or without S1PR1-GFP overexpression were infected with lentivirus for 48 h to express nontarget shRNA (shCtrl) or two different shRNA targeting FLNB (shFLNB_1 and _2). The cells were fixed, stained with anti-S1PR1 antibodies and Alexa Fluor 488–conjugated secondary antibody for endogenous S1PR1 in (*E*), and with DAPI for nuclei, then imaged under a confocal microscope. The scale bar represents 20 μm. Quantification of the fluorescent dot numbers of internalized GFP-tagged S1PR1 in (*C*) and endogenous S1PR1 in (*E*) are shown in (*D*) and (*F*), respectively. Data represent mean ± SEM. ∗∗∗∗*p* < 0.0001 in one-way ANOVA with Bonferroni correction for multiple comparisons test. *G*, HUVECs expressing miniTurbo-tagged S1PR1-WT or -TM4 were incubated with 500 μM biotin for 3 h. Biotinylated proteins were purified by Tamavidin 2-REV magnetic beads. Total (Lysate) and biotinylated (Eluate) FLNB were visualized by a Western blot analysis. *H*, the band intensity in (*G*) was quantified and expressed as a ratio of biotinylated FLNB to total FLNB. Data represent mean ± SEM. ∗∗∗*p* < 0.001 in Student’s *t* test. Data are representatives from at least two independent experiments. Dot numbers were counted in more than 50 cells in each condition from the three sets of independent experiments in (*D*) and (*F*).

We utilized short-hairpin RNA (shRNA) to knock down each of the six candidate proteins and evaluated GFP-tagged S1PR1 localization in HUVECs. As a result, we observed an obvious internalization of S1PR1 after FLNB knockdown but not the other candidates (Fig. 3*A*). We prepared two different shRNA for FLNB and confirmed the efficient FLNB knockdown both in protein level (Fig. 3*B*) and in mRNA level (Fig. S3*A*). No remarkable increase in S1PR1 mRNA was observed with FLNB knockdown in quantitative PCR analysis (Fig. S3*B*), demonstrating that the increased intracellular S1PR1 was not due to an upregulation of S1PR1. Quantification of intracellular fluorescent dot numbers confirmed that FLNB knockdown by two different shRNA significantly promoted S1PR1 internalization in HUVECs overexpressing S1PR1-GFP (Fig. 3, *C* and *D*). Immunostaining of endogenous S1PR1 confirmed the increased internalization after FLNB knockdown (Fig. 3, *E* and *F*). FLNB was detected to the same extent in HUVECs overexpressing S1PR1-WT or -TM4 in the whole cell lysates after the miniTurbo reaction, whereas biotinylated FLNB was more abundant in S1PR1-WT (Fig. 3, *G* and *H*), validating the results from the proteomic analyses.

**Figure 4:**
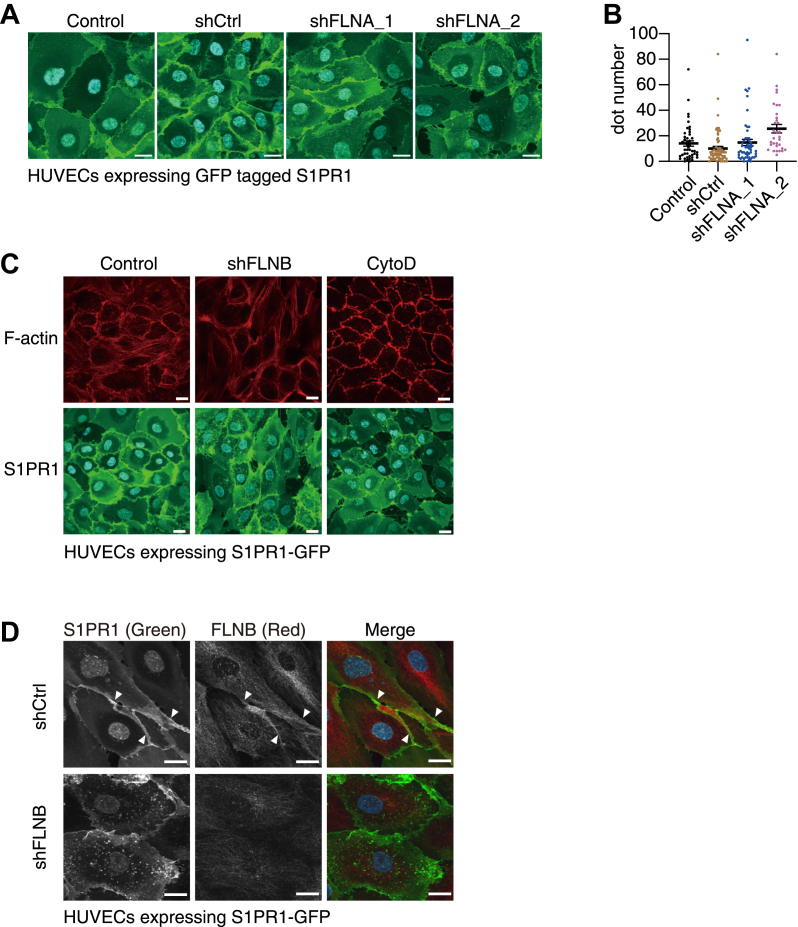
**FLNB induces S1PR1 internalization independent of F-actin network.***A*, representative images of human umbilical vein endothelial cells (HUVECs) showing the localization of GFP-tagged S1PR1 in HUVECs with or without FLNA knockdown. The scale bar represents 20 μm. *B*, quantification of the fluorescent dot numbers in (*A*). Data represent mean ± SEM. *C*, F-actin staining in HUVECs with or without FLNB knockdown. HUVECs expressing GFP-tagged S1PR1 were treated with 1 μM cytochalasin D (Cyto D) for 2 h, fixed and stained with Alexa Fluor 594–conjugated phalloidin for F-actin and with DAPI for nuclei, then imaged under a confocal microscope. The scale bar represents 20 μm. *D*, FLNB staining in HUVECs with or without FLNB knockdown. The cells were fixed, stained with anti-FLNB antibodies and Alexa Fluor 568–conjugated secondary antibody for FLNB and with DAPI for nuclei, then imaged under a confocal microscope. The scale bar represents 20 μm. Data are representatives from at least two independent experiments. Dot numbers were counted in more than 50 cells in each condition from the three sets of independent experiments in (*B*).

Among three filamin family proteins (Filamin A-C), Filamin A (FLNA) is the most studied subtype (32) and has been reported to regulate endocytosis and trafficking of some G protein–coupled receptors (GPCRs) (33). However, FLNA knockdown by shRNA did not alter the localization of S1PR1 (Fig. 4, *A* and *B*). In addition, FLNB knockdown did not change the expression levels of FLNA (Fig. S3*C*). These results indicate that FLNA and FLNB are functionally independent regarding S1PR1 localization in endothelial cells. Since filamin family proteins are reported to play roles in F-actin cross-linking (32), we next examined if the increased internalization of S1PR1 after FLNB knockdown was due to the disruption of the F-actin network. Complete disruption of F-actin by cytochalasin D (an inhibitor of actin polymerization) treatment did not induce S1PR1 internalization (Fig. 4*C*), indicating that S1PR1 internalization after FLNB knockdown was not secondary to major disruption in the F-actin network. Consistent with the F-actin cross-linking role, most of FLNB showed intracellular localization along with the F-actin network, and also showed close localization to S1PR1 in some of the cell cortex areas (Fig. 4*D*, triangles). Together, these results revealed FLNB as a previously unidentified regulator of S1PR1 localization in endothelial cells.

### FLNB knockdown–induced S1PR1 internalization requires ligand binding and receptor phosphorylation

**Figure 5:**
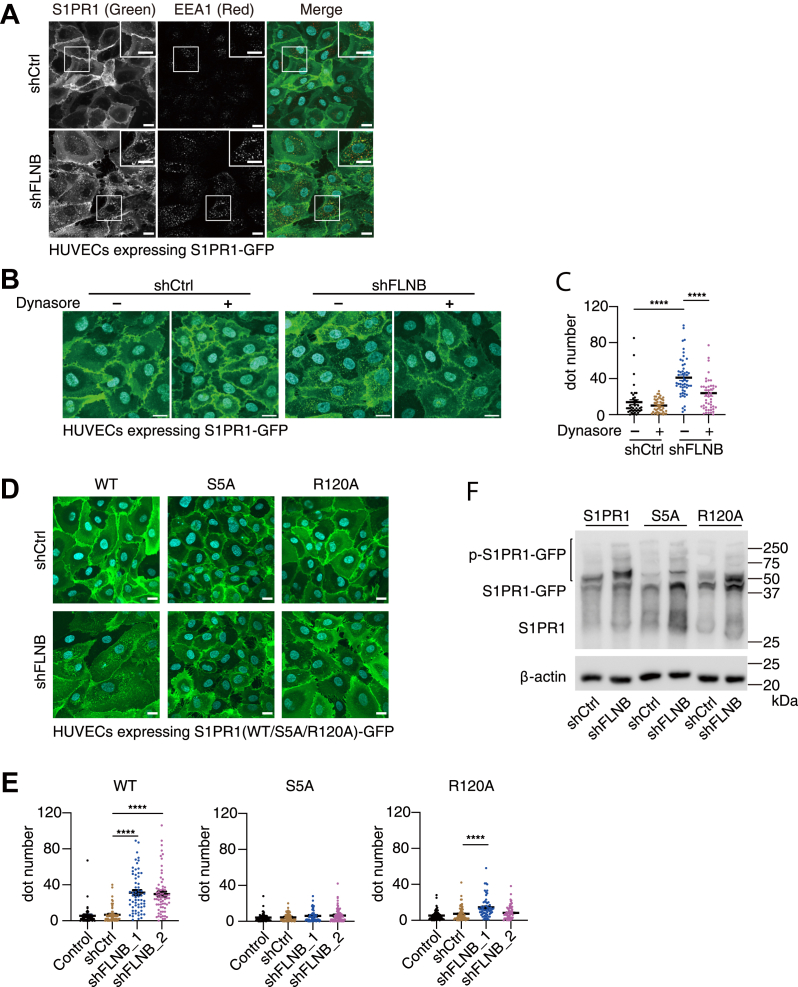
**FLNB knockdown-induced S1PR1 internalization requires ligand binding and receptor phosphorylation.***A*, representative images of human umbilical vein endothelial cells (HUVECs) expressing GFP-tagged S1PR1 with FLNB knockdown. The cells were fixed and stained with anti-EEA1 antibodies and Alexa Fluor 568–conjugated secondary antibody. The images in the white rectangles are enlarged in the insets. Yellow puncta in the merge image indicate colocalization. The scale bar represents 20 μm. *B*, representative images of HUVECs with or without FLNB knockdown in the presence or absence of 80 μM dynasore for 3 h. The scale bar represents 20 μm. *C*, quantification of the fluorescent dot numbers in (*B*). Data represent mean ± SEM. ∗∗∗∗*p* < 0.0001 in one-way ANOVA with Bonferroni correction for multiple comparisons test. *D*, representative images of HUVECs expressing GFP-tagged S1PR1-WT/-S5A/-R120A with or without FLNB knockdown. The scale bar represents 20 μm. *E*, quantification of the fluorescent dot numbers in (*D*). Data represent mean ± SEM. ∗∗∗∗*p* < 0.0001 in one-way ANOVA with Bonferroni correction for multiple comparisons test. *F*, cell lysates were prepared from HUVECs expressing GFP-tagged S1PR1-WT/-S5A/-R120A with FLNB knockdown, and phosphorylated GFP-tagged S1PR1 was separated by phos-tag SDS-PAGE and detected by anti-S1PR1 antibody. Note that molecular weight markers do not indicate the actual sizes due to the phos-tag contained in the gel. Data are representatives from at least two independent experiments. Dot numbers were counted in more than 50 cells in each condition from the three sets of independent experiments in (*C*) and (*E*).

Ligand-activated S1PR1 follows the canonical route of endocytosis for GPCR, which is guided into early endosomes for further sorting (25) and requires GPCR kinase 2–dependent phosphorylation and endocytic regulators such as β-arrestin, clathrin, and dynamin (34). We immunostained early endosomes using an anti-EEA1 antibody and found that most of the internalized S1PR1 after FLNB knockdown overlapped with EEA1-positive endosomes (Fig. 5*A*). Furthermore, the internalized S1PR1 with FLNB knockdown recovered the cell-surface localization after treatment with dynasore (a dynamin inhibitor) for 3 h (Fig. 5, *B* and *C*). These results indicate that the FLNB knockdown–induced S1PR1 internalization utilizes the canonical endocytosis pathway *via* clathrin-coated pits.

Next, we examined whether the S1PR1 internalization induced by FLNB knockdown requires ligand binding and receptor phosphorylation, utilizing the S1PR1 mutant S5A in which five serine residues in the C-terminal region are all mutated to alanine (25), and the R120A mutant, which is deficient in its ability to bind S1P (35). The S5A mutant did not internalize at all after FLNB knockdown, and the R120A mutant showed some internalization but to a much less extent than the WT control (Fig. 5, *D* and *E*). A phos-tag SDS-PAGE followed by Western blot analysis using anti-S1PR1 antibody revealed that FLNB knockdown induced the upward mobility shift of the S1PR1-GFP bands, which indicated the more phosphorylation in multiple sites, while most of these shifted bands were not observed in the S5A mutant and to a lesser extent in the R120A mutant (Fig. 5*F*). These results demonstrate that S1PR1 internalization induced by FLNB knockdown is partially ligand dependent and associated with higher S1PR1 phosphorylation in the C-terminal region, which is required for further steps of the endocytic mechanism.

### FLNB specifically regulates the internalization of S1PR1 in endothelial cells

**Figure 6:**
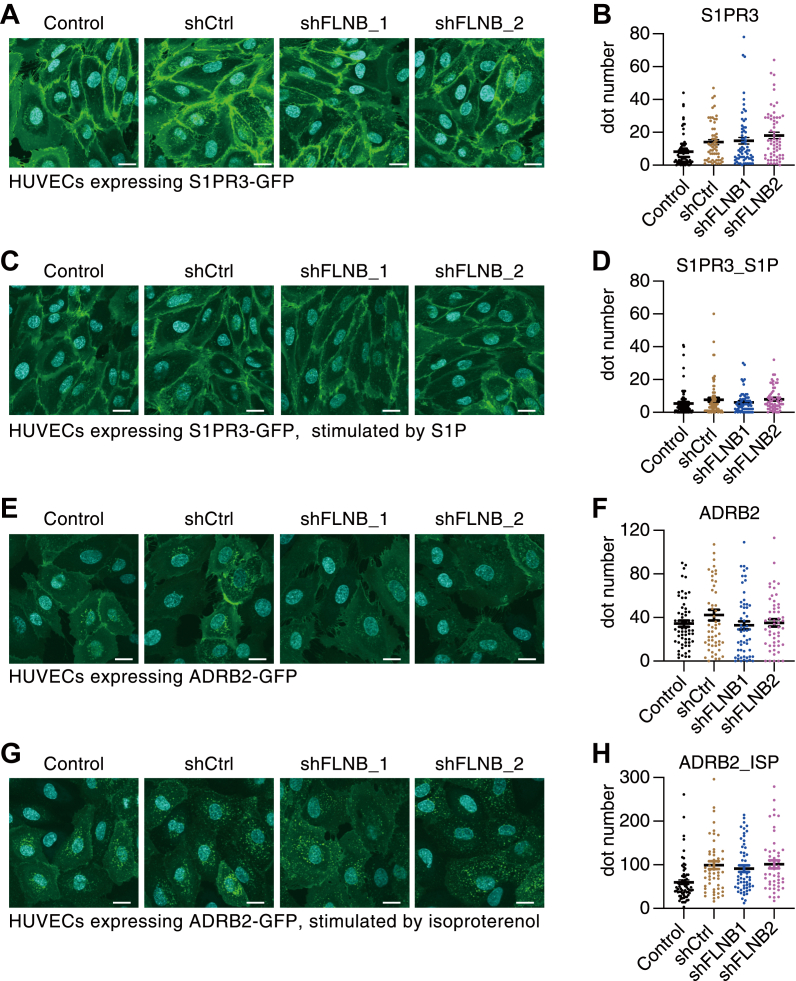
**FLNB specifically regulates the internalization of S1PR1 in endothelial cells.** Representative images of human umbilical vein endothelial cells (HUVECs) expressing GFP-tagged S1PR3 (*A* and *C*) or ADRB2 (*E* and *G*) with FLNB knockdown. The cells were stimulated by 200 nM S1P for 1 h (*C*) or by 10 μM ADRB2-specific agonist isoproterenol for 30 min (*G*). The scale bar represents, 20 μm. Quantification of the fluorescent dot numbers is shown in (*B* and *D*) (S1PR3) and (*F* and *H*) (ADRB2). Data represent mean ± SEM. Dot numbers were counted in more than 50 cells in each condition from the three sets of independent experiments in (*B*, *D*, *F*, and *H*).

S1PR3 is another subtype of S1P receptors expressed in endothelial cells. FLNB knockdown did not induce the internalization of GFP-tagged S1PR3 in HUVECs (Fig. 6, *A* and *B*). Regardless of the FLNB expression level, S1PR3 did not show internalization even after S1P stimulation (Fig. 6, *C* and *D*). We also expressed GFP-tagged β2-adrenergic receptor (ADRB2), a typical GPCR that undergoes ligand-induced endocytosis *via* clathrin-coated pits. FLNB knockdown did not induce ADRB2 internalization, either (Fig. 6, *E* and *F*) and did not have any effects on isoproterenol-induced ADRB2 internalization (Fig. 6, *G* and *H*). Furthermore, we expressed GFP-tagged S1PR1 in HeLa cells in which FLNB is endogenously expressed at a level comparable with that in HUVECs (Fig. S4*A*) (36). However, FLNB knockdown failed to induce S1PR1 internalization in HeLa cells, while S1P stimulation induced the internalization as expected (Fig. S4*B*). These results indicate that the regulation of the receptor endocytosis by FLNB is specific to S1PR1 in endothelial cells.

### FLNB knockdown promotes S1PR1 endocytosis and delays recycling back to the cell surface

**Figure 7:**
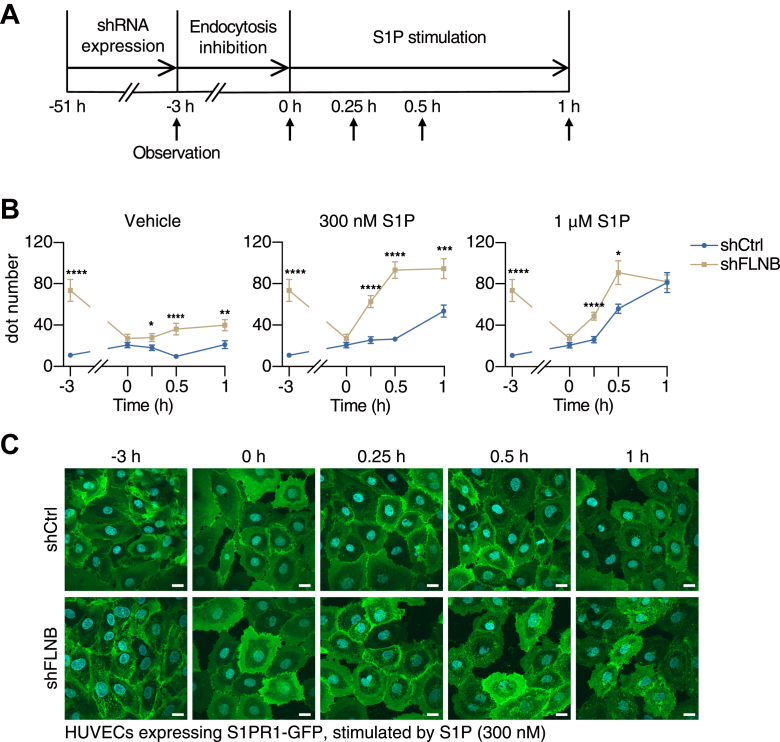
**FLNB knockdown promotes S1PR1 endocytosis.***A*, schematic diagram illustrating the S1PR1 internalization experiment. *B*, quantification of the fluorescent dot numbers in human umbilical vein endothelial cells (HUVECs) expressing GFP-tagged S1PR1 with or without FLNB knockdown. Data represent mean ± SEM. ∗*p* < 0.05, ∗∗*p* < 0.01, ∗∗∗*p* < 0.001, ∗∗∗∗*p* < 0.0001 in Student’s *t* test at the indicated time points. *C*, representative images from the S1PR1 internalization experiment with 300 nM S1P stimulation. The scale bar represents 20 μm. Dot numbers were counted in more than 50 cells in each condition from the three sets of independent experiments in (*B*).

Since FLNB knockdown resulted in the exaggerated S1PR1 internalization, we hypothesized that FLNB maintains S1PR1 on the cell surface of endothelial cells, which are continuously exposed to the high concentration of S1P in blood. To validate this idea, we first inhibited endocytosis by dynasore to keep most of the S1PR1 on the cell surface, then treated the cells with various concentrations of S1P (Fig. 7*A*). Even without S1P stimulation, S1PR1 showed a gradual internalization in FLNB knockdown cells but not in control (Fig. 7*B*, vehicle). With S1P stimulation, S1PR1 got internalized faster and much more in HUVECs with FLNB knockdown compared with control (Fig. 7, *B* and *C*). These results indicate that FLNB functions to keep S1PR1 less sensitive to ligand-induced internalization.

**Figure 8:**
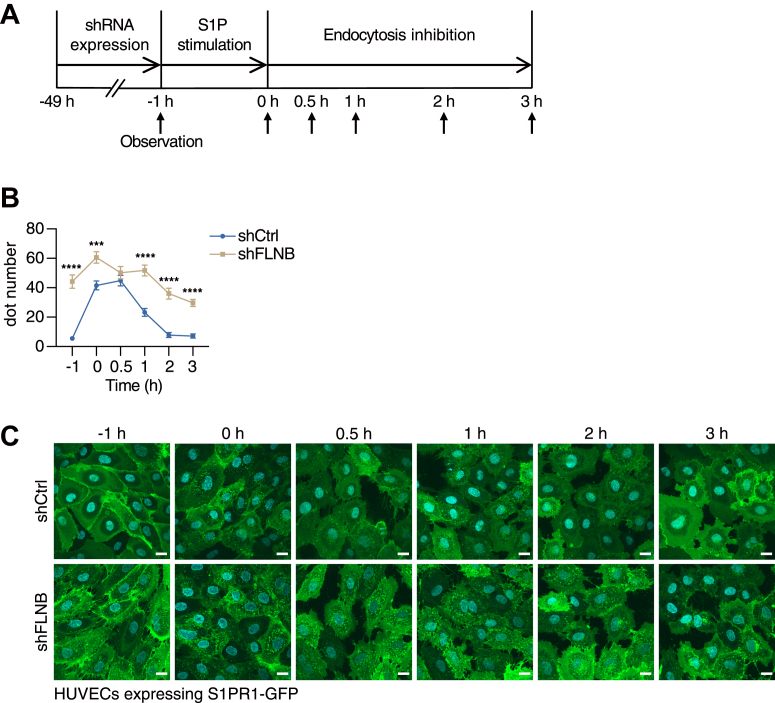
**FLNB knockdown delays recycling back of endocytosed S1PR1 to the cell surface.***A*, schematic diagram illustrating the S1PR1 recycling back experiment. *B*, quantification of the fluorescent dot numbers in human umbilical vein endothelial cells (HUVECs) expressing GFP-tagged S1PR1 with or without FLNB knockdown. Data represent mean ± SEM. ∗∗∗*p* < 0.001, ∗∗∗∗*p* < 0.0001 in Student’s *t* test at the indicated time points. *C*, representative images from the S1PR1 recycling back experiment. The scale bar represents 20 μm. Dot numbers were counted in more than 50 cells in each condition from the three sets of independent experiments in (*B*).

Previous studies have demonstrated that FLNA facilitates the recycling back of endocytosed chemoattractant receptor 2 (CCR2B) from recycling endosomes to the cell surface (37). Thus, we next examined whether FLNB contributes to the recycling back of internalized S1PR1 to the cell surface. HUVECs were stimulated with a high concentration of S1P to induce a massive S1PR1 internalization, then treated with dynasore to inhibit further endocytosis (Fig. 8*A*). As a result, S1PR1 exhibited much slower recycling back with FLNB knockdown (Fig. 8, *B* and *C*). Most of the internalized S1PR1 recycled back to the cell surface in the control cells after 3 h of the dynasore treatment, while there were still many S1PR1 remaining inside the cells with FLNB knockdown, indicating that FLNB knockdown delayed the S1PR1 trafficking from recycling endosomes to the cell surface. Collectively, these results demonstrate that FLNB maintains S1PR1 on the cell surface of HUVECs, not only by keeping S1PR1 less sensitive to ligand-induced internalization but also by facilitating the S1PR1 trafficking from recycling endosomes to the cell surface.

### FLNB knockdown suppresses S1PR1-mediated migration of HUVECs

**Figure 9:**
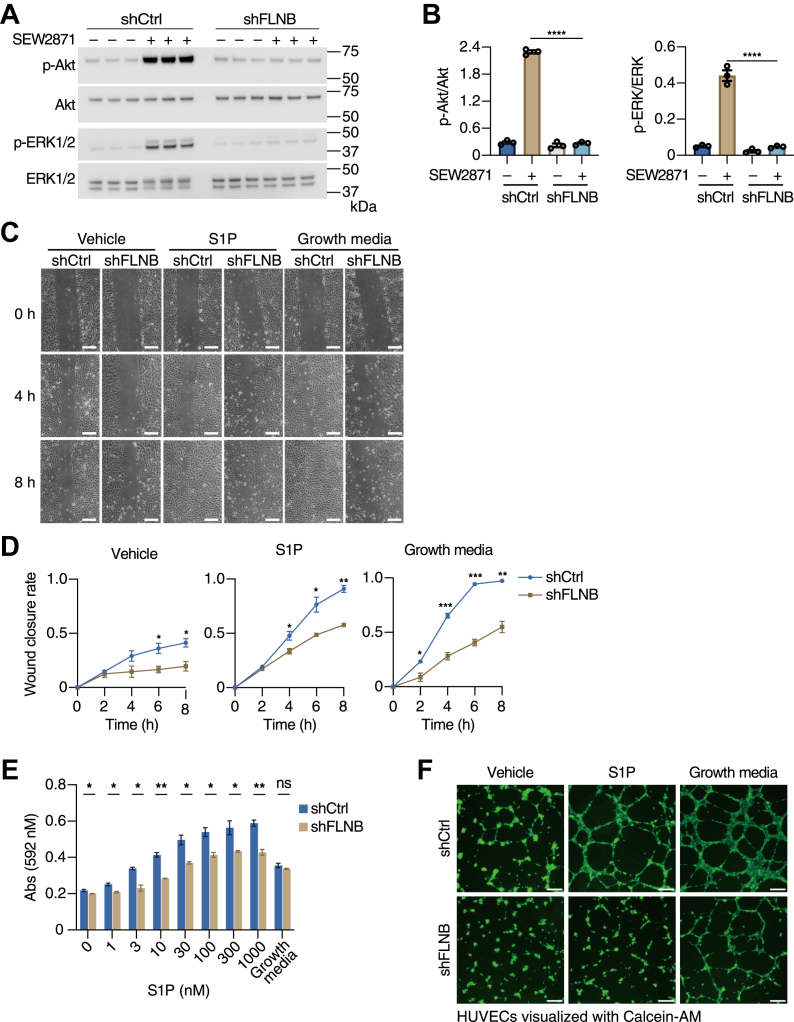
**FLNB knockdown suppresses S1PR1-mediated migration of human umbilical vein endothelial cells (HUVECs).***A*, HUVECs with or without FLNB knockdown were serum-starved for 6 h, then stimulated with S1PR1-specific agonist SEW2871 (1 μM) for 5 min. Total and phosphorylated Akt and ERK1/2 were revealed by Western blot analyses using specific antibodies. *B*, the band intensity in (*A*) was quantified and expressed as a ratio of the phosphorylated forms to the total amounts. Data represent mean ± SEM. ∗∗∗∗*p* < 0.0001 in one-way ANOVA with Bonferroni correction for multiple comparisons test. *C*, representative images from the wound healing assays of HUVECs with or without FLNB knockdown. After the scratch of the HUVEC monolayer, the cells were treated with or without 500 nM S1P or with the growth medium as a positive control. The scale bar represents 200 μm. *D*, the wound closure rates in (*C*) were calculated as follows: (the initial wound area – the wound area at indicated times) divided by the initial wound area. Data represent mean ± SEM. ∗*p* < 0.05, ∗∗*p* < 0.01, ∗∗∗*p* < 0.001 in Student’s *t* test. *E*, Boyden chamber chemotaxis assays of HUVECs with or without FLNB knockdown. HUVECs were seeded into the upper wells of a Boyden chamber and treated with indicated concentrations of S1P or the growth medium added in the lower wells. Upper wells were separated from lower wells by a fibronectin-coated polycarbonate filter with 8-μm pores. After 5-h incubation, the migrated cells on the lower side of the filter were fixed and stained with 0.2% crystal violet, and the absorbance at 592 nm was measured. Data represent mean ± SEM. ∗*p* < 0.05, ∗∗*p* < 0.01 in Student’s *t* test. *F*, HUVECs with or without FLNB knockdown were seeded on the beds of Matrigel in the presence or absence of 500 nM S1P (the growth medium as a positive control) and allowed to migrate to form tubular structures for 5 h. The viable cells were visualized with Calcein-AM. Representative fluorescent images from three independent experiments are shown. The scale bar represents 4 μm.

S1PR1 signaling activates Akt and ERK1/2 through Gαi-mediated pathways (16). To analyze the effects of FLNB knockdown on S1PR1-mediated signaling pathways, we quantified the activation of Akt and ERK1/2 by detecting the phosphorylated forms. To rule out the activation of Akt and ERK1/2 by S1PR3, which is also expressed in HUVECs, we used SEW2871, an S1PR1-specific agonist. As a result, the decrease of cell-surface S1PR1 with FLNB knockdown resulted in less activation of Akt and ERK1/2 in HUVECs (Fig. 9, *A* and *B*).

It has been shown that the activation of S1PR1 induces endothelial cell migration and tube formation (38), which are attenuated by the receptor endocytosis (39). In wound healing assays, S1P stimulation promoted cell migration and wound closure, whereas it was significantly impaired in the FLNB-knockdown cells (Fig. 9, *C* and *D*). Consistent with this, HUVECs with FLNB knockdown exhibited compromised migration ability when exposed to various concentrations of S1P in Boyden chamber chemotaxis assays (Figs. 9*E* and S5). To further characterize the impact of FLNB knockdown on angiogenesis induced by S1P stimulation, tube formation assays were performed by seeding HUVECs on a 3D Matrigel bed. HUVECs showed a tube formation response with longer capillary-like extensions and more complete networks with S1P stimulation, which were completely abrogated when FLNB was knocked down (Fig. 9*F*). Together, these results demonstrate that the loss of S1PR1 from the cell surface by FLNB knockdown compromises the activation of signaling molecules such as Akt and ERK1/2, migration, and morphogenetic responses after S1P stimulation.

### FLNB knockdown impairs the vascular barrier function

**Figure 10:**
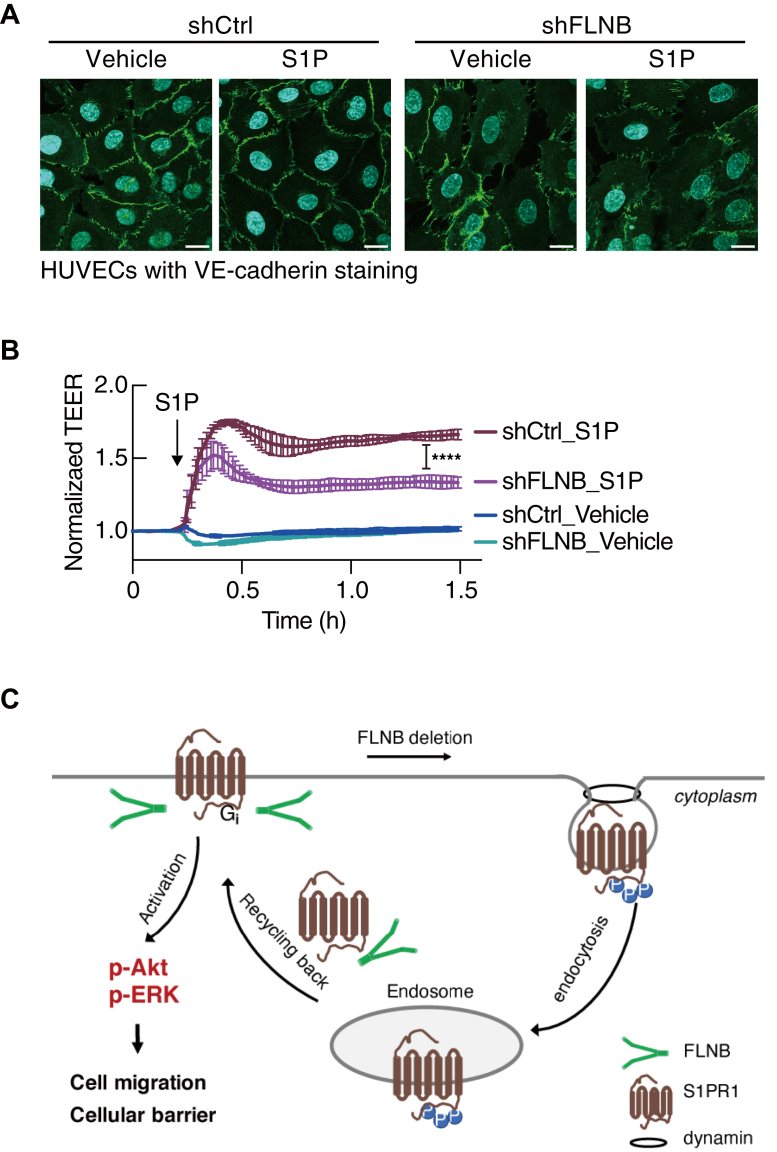
**FLNB knockdown impairs the vascular barrier function.***A*, VE-cadherin immunostaining in human umbilical vein endothelial cells (HUVECs) with or without FLNB knockdown in the presence or absence of 300 nM S1P treatment for 30 min. The scale bar represents 20 μm. Representative fluorescent images from three independent experiments are shown. *B*, trans-endothelial electrical resistance (TEER) was monitored in the confluent monolayers of HUVECs with or without FLNB knockdown (n = 4). S1P (100 nM) was added at the time point indicated by an arrow. Data represent mean ± SEM. ∗∗∗∗*p* < 0.0001 in one-way ANOVA followed by Bonferroni correction for multiple comparisons test. *C*, graphical abstract illustrating that FLNB is important for maintaining S1PR1 cell-surface residency.

S1PR1 signaling strengthens adherens junctions *via* VE-cadherin (40) and thus regulates the vascular barrier function (41). Immunostaining of VE-cadherin revealed that S1P-induced assembly of adherens junctions was largely impaired in HUVECs with FLNB knockdown, while VE-cadherin showed continuous and zipper-like structures in the control cells after S1P stimulation (Fig. 10*A*). To further confirm this, the vascular barrier function was monitored by measuring trans-endothelial electrical resistance. As expected, S1P stimulation of the control HUVEC monolayer induced a sustained increase in trans-endothelial electrical resistance, which means enhanced vascular barrier integrity (Fig. 10*B*), and FLNB knockdown obviously attenuated this increase. These results suggest that FLNB plays an important role in maintaining S1PR1 on the cell surface, enabling sustained endothelial responses to S1P, and thereby enhances the barrier integrity of the endothelial monolayer.

## Discussion

S1P is contained in blood and lymph at the concentration above the equilibrium dissociation constant (K_d_) of S1PR1 (∼10 nM); thus, S1PR1 is almost completely internalized in circulating lymphocytes while in blood and lymph (42). Moreover, previous studies have demonstrated that CD69 binds to S1PR1 and induces its internalization in interferon-activated lymphocytes, which enables a rapid adaptive immune response (29, 31). Exposed to the same concentration of S1P in blood, endothelial S1PR1 mainly localizes on the cell surface. However, it is not known how the cell surface retention of S1PR1 is achieved in endothelial cells. Our study identified FLNB as a novel regulator of S1PR1 in endothelial cells, which sustains the cell-surface retention of S1PR1 not only by decreasing ligand- and phosphorylation-dependent endocytosis of S1PR1 but also by facilitating the recycling back of endocytosed S1PR1 to the cell surface. Our data demonstrate that FLNB ablation induces S1PR1 internalization and attenuates downstream signaling of ligand-activated S1PR1, thereby impairing S1PR1-mediated cellular functions such as migration and vascular barrier integrity (Fig. 10*C*).

FLNB is a cytoplasmic F-actin-binding protein consisting of an actin-binding domain, 24 immunoglobulin-like repeat domains and 2 hinges. FLNB is highly expressed in endothelial cells and skeletal muscles, and FLNB deletion in embryos led to impaired development of microvasculature and skeletal system (43). Studies have also shown that FLNB contributes to cell migration in various cell types (44, 45, 46), although there was no report showing its relationship with S1PR1. FLNA is the first-identified isotype of the filamin family and functions as a cross-linking protein of actin filaments (32). Substantial studies have demonstrated its implications in transmembrane receptor dynamics (47) either by anchoring receptors to F-actin (43) or directly regulating receptor trafficking (48). In addition, FLNA has been reported to colocalize with S1PR1 as well as sphingosine kinase 1 at membrane ruffles to orchestrate cell migration (49). FLNA and FLNB share about 70% overall amino acid identity (50) and function as actin-binding proteins, which organize the actin cytoskeleton and maintain the connections with extracellular matrices, thereby stabilizing the plasma membrane (51). However, FLNA knockdown did not induce S1PR1 internalization in endothelial cells in our study, indicating a specific effect of FLNB on S1PR1. Interestingly, the ability of FLNB to maintain cell-surface S1PR1 localization appears to be independent of its F-actin cross-linking function, as actin depolymerization by cytochalasin D failed to induce S1PR1 internalization. FLNB regulates cell-surface S1PR1 localization in a receptor- and endothelial cell–specific manner, demonstrated by the facts that S1PR3 or ADRB2 localization was not affected by FLNB knockdown in endothelial cells and that S1PR1 maintains cell-surface residency in HeLa cells after FLNB knockdown.

Although we demonstrated that S1PR1 was inclined to internalize without FLNB support in a canonical GPCR endocytosis pathway (ligand- and receptor phosphorylation–dependent and dynamin-mediated sorting to early endosomes), the molecular mechanisms still remain to be clarified. The results from the phos-tag SDS-PAGE analysis showed that FLNB knockdown led to more phosphorylation of S1PR1 specifically in the C-terminal region (Fig. 5*F*). The interaction of FLNB with S1PR1 might prevent S1PR1 from the phosphorylation by G protein–coupled receptor kinases. FLNA has been reported to function also as a receptor scaffold. FLNA deletion specifically prevents the loading of chemokine receptor CCR2B and ADRB2 onto the actin-enriched microdomains and delays their recycling back to the cell membrane (37). This raises another possibility that FLNB might work as a cargo adaptor maintaining the cell-surface residency of S1PR1 and promoting its recycling back through actin-enriched microdomains.

A previous study has found that FLNB-knockout embryos developed impaired microvascular structures, which was ascribed to the reduced capability of endothelial migration (52). Although FLNB has been demonstrated to be involved in endothelial migration by regulating the interaction of EGFR with Rac-1 and Vav-2 (45), the detailed mechanisms for FLNB involvement in cellular migration remain unknown. Our study showed that FLNB knockdown impaired endothelial cell migration most likely by inducing S1PR1 internalization and making endothelial cells less sensitive to S1P, one of the most potent chemoattractants of endothelial cells. Although further *in vivo* studies are required, impaired microvascular development in FLNB knockout mice might be ascribed to impaired S1PR1 functions due to the failure of cell-surface localization.

Limitations of our study include the uncertainty about the transferability of the regulatory effects of FLNB on S1PR1 signaling to clinical applications and demonstration of the interaction between FLNB and S1PR1 *in vivo*. In physiological conditions, endothelial S1PR1 plays roles in maintaining vascular homeostasis, such as regulations of vascular development, permeability, and inflammation (21, 53). However, in the tumor microenvironment, S1PR1 promotes cancer progression by enhancing tumor vascularization and reducing hypoxia (19). Inhibition of S1PR1 expressed in tumor vessels effectively reduces angiogenesis and delays tumor growth *in vivo* (19). Fingolimod, a functional antagonist of S1PR1 and approved as an oral drug for treating relapsing forms of multiple sclerosis, has been reported to exhibit anticancer properties by inhibiting the S1PR1 signaling pathways in various types of cancer (54, 55, 56). However, the clinical application of fingolimod is currently limited by its immune suppression effects. Fingolimod exerts its effects by inducing S1PR1 internalization and subsequent degradation in proteasomes (57). Our study revealed that FLNB suppression also induces S1PR1 internalization. It could be interesting to examine if fingolimod and FLNB suppression have a synergistic effect to abrogate S1PR1 functions for the inhibition of tumor angiogenesis.

In summary, we provided the evidence that FLNB functions as a novel regulator of S1PR1 in endothelial cells. Our data demonstrate that FLNB is important to maintain cell-surface residency of S1PR1, thus enabling S1PR1 to fulfill proper endothelial functions such as cell migration and vascular permeability. Endothelial S1PR1 shows intracellular localization in the inflammation-prone areas of aorta, in contrast to cell-surface accumulation under the laminar flow (12). Targeting FLNB to regulate S1PR1 localization may provide additional therapeutic interventions for treating vascular inflammation and related vascular diseases as well as tumor angiogenesis.

## Experimental procedures

### Cell culture and reagents

HUVECs and human dermal microvascular endothelial cells were cultured in EGM-2 (Lonza) supplemented with 10% (v/v) fetal bovine serum (Gibco). HUVECs and human dermal microvascular endothelial cells with passage number 4 to 7 were used for the experiments. Mouse embryonic endothelial cells were cultured in M199 medium supplemented with 10% fetal bovine serum. HEK293, HEK293T, and HeLa cells were cultured in Dulbecco’s modified Eagle’s medium (Thermo Fisher Scientific) supplemented with 10% fetal bovine serum and penicillin–streptomycin (Corning). CHO cells were cultured in Ham's F-12 nutrient mixture (Gibco). All cells were maintained in a humidified atmosphere under 5% CO_2_.

Antibodies against Akt (#9272), phospho-Akt (Ser473, #9271), ERK1/2 (#9102), phospho-ERK1/2 (T202/Y204, #9106), and S1PR1 (#63335) were purchased from Cell Signaling Technology. Antibodies against β-actin (#A5316) and (−)-Isoproterenol hydrochloride (#I6504) were from Sigma-Aldrich. Antibodies against Filamin B (#AB9276) were from Merck. Streptavidin-HRP (#21130) was from Thermo Fisher Scientific. SEW2871 (#10006440) and Cytochalasin D (#11330) were from Cayman Chemical. Calcein-AM (#349-07201) was from Dojindo. Matrigel GFR (#354230) was from Corning. Dynasore (#ab120192) was from Abcam.

### Plasmid DNA constructs

Plasmids encoding S1PR1-WT/-TM4/-R120A/-S5A-GFP were reported previously (25, 29, 35). TurboID (Addgene, #107169) and miniTurbo (Addgene, #107170) fragments were amplified by PCR and subcloned into pLVX-TetOne-Puro vector (Takara Bio, #631847), which had been inserted with the S1PR1-WT/-TM4-GFP fragment, to replace GFP with the TurboID or miniTurbo fragment. The shRNA-targeted sequences were listed in the Supporting information (Table S1). For the constructions of plasmids to express shRNA against target genes, double-stranded oligonucleotides were cloned into the pLKO.1-TRC vector (Addgene, #10878). A nonsense scrambled oligonucleotide was used as a negative control. All of the inserted DNA fragments were confirmed by performing DNA sequencing.

### Lentivirus-mediated stable or transient expression of the constructs in HUVECs

HEK293T cells were transfected with the constructed plasmids along with lentiviral packaging plasmids pVSV-G, pMDL/pPRE, and pRSV-REV (Addgene) using a calcium phosphate method. The lentiviral-containing media were collected 72 h after the transfection, filtered through a 0.45-μm filter, then aliquoted and stored at −130 °C until use. HUVECs were infected with packaged lentivirus for the expression of the constructs (S1PR1-WT-GFP/-TurboID/-miniTurbo, S1PR1-TM4-GFP/-TurboID/-miniTurbo, S1PR3/ADRB2-GFP or shRNAs). After incubation for 48 h, the cells were used for transient experiments or selected with 2 μg/ml puromycin (Thermo Fisher, #A1113803) for more than a week for stable expression.

### Western blotting

HUVECs were lysed in a cell lysis buffer (50 mM Tris-HCl [pH 8.0], 100 mM NaCl, 2 mM EDTA, 10 mM β-glycerophosphate, 1 mM Na_3_VO_4_, 5 mM NaF, 1% Triton X-100, and 0.5% Fos-Choline) and protease inhibitor cocktail (Roche, #11873580001). Protein concentration was determined by Pierce BCA Protein Assay Kit (Thermo Fisher, #23225), and equal amounts of proteins were loaded onto NuPAGE Novex 4%-12% Bis-Tris gels (Invitrogen, #NP0323BOX) or phos-tag SDS-PAGE gels. After electrophoresis, the phos-tag gels were incubated in running buffer (0.1 M Tris base, 0.1 M Mops, 0.1% SDS, 1 mM Sodium Bisulfite) containing 10 mM EDTA for 10 min three times to remove metal ion, then in transfer buffer (25 mM Tris, 192 mM Glycine, 20% (v/v) methanol) for 10 min. Subsequently, the gels were electroblotted to the polyvinylidene difluoride membrane (Invitrogen, #LC2005). The membrane was incubated in 5% (w/v) skim milk in Tris-buffered saline containing 0.1% Tween 20 (TBS-T) for 1 h followed by incubation with the primary antibody in 5% (w/v) skim milk overnight. After washing with TBS-T 3 times, the membrane was incubated with HRP-conjugated secondary antibody. To detect immunoblot signals, the membrane was incubated with Western HRP Substrate (Millipore, #WBLUF0100) and visualized in an ImageQuant LAS 4010 system (GE Healthcare).

### Proximity labeling with a TurboID system

HUVECs were treated with 100 ng/ml doxycycline for 24 h to induce S1PR1-WT/-TM4-miniTurbo expression, then treated with 500 μM biotin at 37 °C for 3 h. The biotinylation reaction was terminated with ice-cold PBS. The cells were lysed using a phase transfer surfactant (PTS) buffer (58) containing 100 mM Tris-HCl (pH 9.0), 12 mM sodium deoxycholate, 12 mM sodium lauroyl sarcosinate, and protease inhibitor cocktail. Protein concentration was determined by using the BCA protein assay kit.

### Purification of biotin-labeled proteins and mass spectrometer analysis

Tamavidin 2-REV magnetic beads (Wako pure Chemicals, #133-18611) were added to the cell lysate (50 μl beads suspension for 200 μg proteins) in the PTS buffer (pH 7.4) to capture the biotinylated proteins and incubated overnight at 4 °C. The beads were washed and recovered in the PTS buffer (pH 7.4) using a magnetic stand. The biotinylated proteins captured on the beads were digested by trypsin and LysC and analyzed by liquid chromatography–tandem mass spectrometry. In detail, the proteins on the beads were incubated with 10 mM dithiothreitol (Wako pure Chemicals, # 040-29222) in 50 mM ammonium bicarbonate solution for 30 min at room temperature, then incubated with 50 mM iodoacetamide (Wako pure Chemicals, # 093-02152) for 30 min at room temperature in the dark. The reaction was terminated by adding 4 volumes of 50 mM ammonium bicarbonate solution. The proteins on the beads were digested with 0.5 μg trypsin (Promega, #V5280) and 0.1 μg LysC (Wako pure Chemicals, #125-05061) overnight at 37 °C. Sodium deoxycholate and sodium lauroyl sarcosinate in the PTS buffer were removed by ethyl acetate extraction after the acidification of the samples by formic acid. The digested peptides were desalted with C18 stage GL-tips (GL Sciences, #7820-11200), dried up, then resuspended in water containing 0.1% formic acid, and applied to the liquid chromatography–tandem mass spectrometry analysis with an Eksigent Ekspert NanoLC 425 system coupled to a TripleTOF 6600 mass spectrometer (Sciex). The peptide mixture was separated by an ODS column (Eksigent ChromXP-C18-CL, 3 μm, 120 Å, 0.075 mm I.D. × 150 mm L, Sciex) with 2 to 30% acetonitrile gradient containing 0.1% formic acid for 60 min. Protein identification was performed with the Paragon algorithm search engine using a ProteinPilot software (Sciex).

### Quantitative PCR analysis

Gene expression levels were examined by quantitative PCR analyses. Briefly, total RNA was isolated from cells using ISOGEN II (Nippon Gene, #311-07361) and purified using RNeasy Mini Kit (Qiagen). Total RNA (500 ng) was reverse-transcribed to cDNA using ReverTra Ace (Toyobo, #FSQ-101). Quantitative PCR was performed using Thunderbird SYBR Green master mix (Toyobo, #QPS-201) and a StepOne Plus Real-Time PCR System (Thermo Fisher). Primers used for the quantitative PCR analyses are listed in Table S2.

### S1PR1 internalization assay

HUVECs stably expressing S1PR1-GFP were infected for 48 h with lentivirus to induce shRNA-mediated suppression of the target genes, then transferred onto 35-mm glass-bottomed dishes and incubated for another 24 h. The cells were treated as indicated in the figure legends and fixed in methanol. Nuclei were stained with DAPI (PerkinElmer, #CP81). Confocal fluorescence microscopy was performed using a FluoView FV10i system (Olympus). Intracellular S1PR1 dot numbers were quantified by Matlab software (version R2022a, MathWorks Software).

### Immunofluorescent staining

Cells seeded on 35-mm glass-bottomed dishes were fixed with 4% paraformaldehyde for 10 min and blocked in 2% bovine serum albumin for 1 h. Early endosomes were stained with anti-EEA1 antibody (BD Transduction, #610457) and Alexa Fluor 568–conjugated secondary antibodies (Thermo Fisher, #A-11004), S1PR1 was stained with anti-S1PR1 antibody (Cell Signaling Technology, #63335) and Alexa Fluor 488–conjugated secondary antibodies (Thermo Fisher, #A28175), VE-cadherin was stained with anti-VE-cadherin antibody (Santa Cruz Biotechnology, #sc-9989) and Alexa Fluor 488–conjugated secondary antibodies, F-actin was stained with Alexa Fluor 594 phalloidin (Thermo Fisher, #A12381), and nuclei were stained with DAPI. Confocal fluorescence microscopy was performed as mentioned above.

### Wound healing assay

HUVECs were seeded on 12-well plates and cultured to confluency. The HUVEC monolayers were scratched using a sterile 200-μl pipette tip. After scratching, the monolayers were gently washed with warmed PBS to remove cell debris. Subsequently, the cells were treated with 500 nM S1P or complete EGM-2 growth media. The wound closure of the monolayer was imaged at the indicated time by an inverted microscope (Olympus) with a digital camera (Cannon). The wound area was measured by using ImageJ software (version 1.53p). The wound closure rates were calculated as follows: (the initial wound area – the wound area at an indicated time) divided by the initial wound area.

### Chemotaxis assay

Chemotaxis assay was performed using a 96-well chemotaxis chamber system (Neuroprobe, #AB96). After serum starvation in EBM-2 medium for 3 h, HUVECs were seeded in the upper well of the chemotaxis chamber at a density of 1 × 10^5^ cells/well and were allowed to migrate toward chemoattractant (a various concentration of S1P) in the lower well, which was separated from the upper well by a fibronectin-coated polycarbonate filter with 8-μm pores.

### Tube formation assay

Matrigel (100 μl/well) (Corning, #354230) was added to a 96-well plate and allowed to polymerize at 37 °C for 30 min. HUVECs were serum-starved for 3 h, resuspended in medium as indicated, then seeded at 2 × 10^4^ cells/well onto the Matrigel. After 5-h incubation, the viable cells were visualized with Calcein-AM (Dojindo, #349-07201) and imaged by an inverted fluorescent microscope at low magnifications (5 × ) (Leica).

### Measurement of endothelial barrier function

Endothelial barrier function was evaluated by measuring the resistance of a cell-covered electrode in microtiter plates using an xCELLigence Real-Time Cell Analyzer system (ACEA BioSciences) in accordance with the manufacturer’s instructions. Briefly, HUVECs were plated on fibronectin-coated electrodes at a density of 1 × 10^4^ cells/well and allowed to reach confluent monolayers. The cells were starved for 4 h in EBM-2 medium supplemented with 0.1% bovine serum albumin, followed by 100 nM S1P stimulation. Changes in the resistance were monitored and expressed as fractional resistance, normalized to the baseline values before the stimulation with S1P.

### Statistical analysis

Statistical analysis was performed using GraphPad Prism software (version 9, GraphPad Software Inc). To determine the significance among three or more test groups, analysis of variance (ANOVA) was used, followed by Bonferroni test for comparison with the control group or Tukey test to compare all groups. Two-tailed Student’s *t* test was used for the direct comparison of two groups. A *p*-value < 0.05 was considered to be statistically significant. Data were presented as mean ± SEM as indicated in each figure legend. Asterisks were used to indicate distinct *p*-values: ∗*p* < 0.05, ∗∗*p* < 0.01, ∗∗∗*p* < 0.001 and ∗∗∗∗*p* < 0.0001.

## Data availability

All data needed to evaluate the conclusions in the paper are present in the paper or the Supporting information. The plasmid DNA constructs used in this study are available upon request. The mass spectrometry proteomic data are available from the ProteomeXchange Consortium with the accession number PXD039461.

## Supporting information

This article contains supporting information.

## Conflict of interest

The authors declare that they have no conflicts of interest with the contents of this article.
