# Supplementary material for: Actin-binding protein filamin B regulates the cell-surface retention of endothelial sphingosine 1-phosphate receptor 1
Source: J Biol Chem. 2023 May 21;299(7):104851. doi: 10.1016/j.jbc.2023.104851 (PMC10300261; doi:10.1016/j.jbc.2023.104851)
Supplement: Supporting Tables S1 and S2 and Figures S1–S5 [file mmc1.docx]

*Supporting information*

**Actin-binding protein Filamin B regulates the cell-surface retention of endothelial** **sphingosine 1-phosphate receptor 1**

Xian Zhao^1^, Keisuke Kiyozuka^1^, Akimitsu Konishi^1^, Reika Kawabata-Iwakawa^2^, Yoji Andrew Minamishima^1^, Hideru Obinata^3^***

^1^Department of Biochemistry, Gunma University Graduate School of Medicine, Gunma, Japan.

^2^Division of Integrated Oncology Research, Gunma University Initiative for Advanced Research, Gunma, Japan.

^3^Education and Research Support Center, Gunma University Graduate School of Medicine, Gunma, Japan.

*Correspondence to Hideru Obinata, obi@gunma-u.ac.jp

**List of contents**

Table S1. Oligomers for the construction of shRNA lentivirus vectors

Table S2. Primers for qPCR

Fig. S1. S1PR1 mutant S1PR1-TM4 shows intracellular localization in endothelial cells.

Fig. S2. Identification of proximal and interacting proteins of S1PR1 in HUVECs.

Fig. S3. Confirmation of mRNA levels after shRNA-mediated knockdown.

Fig. S4. FLNB knockdown does not promote S1PR1 internalization in HeLa cells.

Fig. S5. FLNB knockdown suppresses S1PR1-mediated migration of HUVECs.

Table S1. Oligomers for the construction of shRNA lentivirus vectors

| Oligomers | Sequence |
| --- | --- |
| HSPG2-sh1 | 5'-CCGGGCTGCCAAGGGACGTATATATCTCGAGATATATACGTCCCTTGGCAGCTTTTTG |
| HSPG2-sh2 | 5'-CCGGCGGTCAAGATTGAGTCCTCATCTCGAGATGAGGACTCAATCTTGACCGTTTTTG |
| ITGA2-sh1 | 5'-CCGGATGGCAATATCACGGTTATTCCTCGAGGAATAACCGTGATATTGCCATTTTTTG |
| ITGA2-sh2 | 5'-CCGGCCGGCCAGATAGTGCTATATACTCGAGTATATAGCACTATCTGGCCGGTTTTTG |
| EF1G-sh1 | 5'-CCGGCAGGTGGACTACGAGTCATACCTCGAGGTATGACTCGTAGTCCACCTGTTTTTG |
| EF1G-sh2 | 5'-CCGGAGCTTTGCTGATTCCGATATACTCGAGTATATCGGAATCAGCAAAGCTTTTTTG |
| FLNB-sh1 | 5'-CCGGGCCTTCAGGAATCGGGATTAACTCGAGTTAATCCCGATTCCTGAAGGCTTTTTG |
| FLNB-sh2 | 5'-CCGGCCTGTGGATAATGCACGAGAACTCGAGTTCTCGTGCATTATCCACAGGTTTTTG |
| FGD5-sh1 | 5'-CCGGGTGCTCCTCACAGACTATTTACTCGAGTAAATAGTCTGTGAGGAGCACTTTTTG |
| FGD5-sh2 | 5'-CCGGCCACCCAGTAATAAACTATTTCTCGAGAAATAGTTTATTACTGGGTGGTTTTTG |
| G3BP2-sh1 | 5'-CCGGGTGATGATCGCAGGGATATTACTCGAGTAATATCCCTGCGATCATCACTTTTTG |
| G3BP2-sh2 | 5'-CCGGCGGGAGTTTGTGAGGCAATATCTCGAGATATTGCCTCACAAACTCCCGTTTTTG |

Table S2. Primers for qPCR

| Primers | Sequence |
| --- | --- |
| HSPG2-forward | 5'-TGAGTCCTTCTACTGGCAGC |
| HSPG2-reverse | 5'-GATGTTGTTGCCCGTGATCTG |
| ITGA2-forward | 5'-GGTGCTCCTCGGGCAAATTA |
| ITGA2-reverse | 5'-GAGCCAATCTGGTCACCTCG |
| EF1G-forward | 5'-CCTTCGCCAGTGTCATCCTT |
| EF1G-reverse | 5'-CCAATCTGGACTCAGCGGAA |
| FLNB-forward | 5'-ACACCAAAGCTGCAGGAAGT |
| FLNB-reverse | 5'-GGCTCTTTGGAATGTGGTGT |
| FGD5-forward | 5'-GAGGGCAGCAGTGAAGTAGG |
| FGD5-reverse | 5'-GTGTGTGTCCGGATGAGTTG |
| G3BP2-forward | 5'-AGAGGTGGTGGTGATGATCG |
| G3BP2-reverse | 5'-AGCCAAGTTTCTGTGCCATG |
| FLNA-forward | 5'-CCATGACAACACCTACACAGTCA |
| FLNA-reverse | 5'-CTTGGAGATACTGCCACTGAGAA |
| S1PR1-forward | 5'-ATCATGGGCTGGAACTGCATCA |
| S1PR1-reverse | 5'-CGAGTCCTGACCAAGGAGTAGAT |
| GAPDH-forward | 5'-GCCAAGGTCATCCATGACAACT |
| GAPDH-reverse | 5'-GAGGGGCCATCCACAGTCTT |
| S1PR3-forward | 5'-ATCCTGCCCCTCTACTCCAAGA |
| S1PR3-reverse | 5'-GTGCGTAGAGGATCACGATGGT |

**Fig. S1. S1PR1 mutant S1PR1-TM4 shows intracellular localization in endothelial cells.** (**A, B**) Representative images of human dermal microvascular endothelial cells (A) and mouse embryonic endothelial cells (B) showing the localization of GFP-tagged S1PR1-WT or -TM4. Scale bar, 20 µm. Data are representatives from at least 2 independent experiments.

**Fig. S2.** Identification of proximal and interacting proteins of S1PR1 in HUVECs**.** (**A**) Diagrams of the expression cassettes used for the expression of a biotin ligase, TurboID. TurboID was fused to the C-terminus of S1PR1-WT/-TM4. Expression was under the control of a TRE3Gs promoter and doxycycline-dependent transactivator protein Tet-On 3G. (**B**) Western blot of S1PR1-WT/-TM4-TurboID expressions in HUVECs induced by different concentrations of doxycycline (Dox) for 24 h. The cells were lysed, and the ligase-tagged S1PR1 were identified by anti-S1PR1 antibody. (**C**) Western blot of the biotinylated proteins by S1PR1-WT/-TM4- TurboID expression in HUVECs. The cells were treated with 100 ng/ml doxycycline for 24 h, then treated with 500 μM biotin for indicated times. The cells were lysed, and the biotinylated proteins were visualized by streptavidin-HRP. Data are representatives from at least 2 independent experiments.


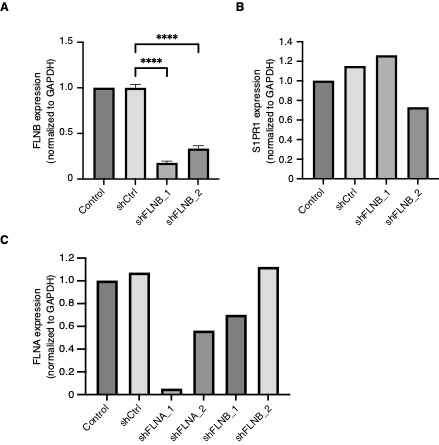


**Fig. S3. Confirmation of mRNA levels after shRNA-mediated knockdown.** (**A**) Expression levels of FLNB mRNA after knockdown by 2 different shRNA in HUVECs. ****, p < 0.0001, one-way ANOVA with Tukey multiple comparisons correction. (**B**) qPCR analysis of S1PR1 mRNA levels in HUVECs with or without FLNB knockdown. (**C**) qPCR analysis of FLNA mRNA levels in HUVECs with FLNA or FLNB knockdown.

**Fig. S4. FLNB knockdown does not promote S1PR1 internalization in HeLa cells.** (**A**) qPCR analysis of FLNB mRNA levels in HUVECs, HeLa and HEK293 cells. (**C**) Representative images of GFP-tagged S1PR1 in HeLa cells with or without FLNB knockdown, or with 100 nM S1P stimulation for 1 h. Scale bar, 20 μm. Data are representatives from at least 2 independent experiments.

**Fig. S5. FLNB knockdown suppresses S1PR1-mediated migration of HUVECs.** Boyden chamber chemotaxis assays in HUVECs with or without FLNB knockdown. HUVECs were seeded into a Boyden chamber and treated with different concentrations of S1P for 5 h. Migrated cells were fixed and stained with 0.2% crystal violet, and the absorbance of each well at 592 nm was measured using a microplate reader. A representative image of the stained cells is shown from more than 3 independent experiments.
